# A Treatise on Therapeutics, Comprising Materia Medica and Toxicology

**Published:** 1874-06-01

**Authors:** 


A TREATISE on Therapeutics, comprising Materia Medica and Toxicology. By H. C. Wood, Jr., M. D., Prof. of Botany, etc., in the Medical Department of the University of Pennsylvania, &c., &c. Philadelphia: Lippincott & Co., 1874.

This work, embodying as it does a vast amount of original research, is an exceedingly valuable contribution to medical literature. It was written "with special reference to the application of the physiological action of drugs to clinical medicine." The idea upon which the work is constructed, is, that a knowledge of the therapeutic effects, and modes of action and application to the cure of disease, of any medicine, may be derived from a knowledge of its physiological operation.

It is hardly within the province of a review to discuss such a proposition. Yet the fallacy of the view may at once be seen, when we consider that there is, in many diseases, a specific element, which a medicine may especially be adapted to remove, and yet without producing any obvious disturbance of physiological processes. No amount of physiological research would enable us to say, that this medicine will cure ague, that rheumatism, and the other syphilis. These clincal facts must be determined by clinical observation.

Much can undoubtedly be told however of the probable effect, or utility of a certain medicine, whose physiological action is well understood, on a disease the nature of which is thoroughly comprehended. Yet we know less of the intimate nature of pathological processes, than we do of the modes of action of medicines. And it seems to us unwise to build a work on so insecure a foundation.

Of course the author has adopted a new classification of drugs. Every author does. And many seem to consider the elaboration of a new classification sufficient justification for the publication of a new book. But the present book is one the physician and student cannot well afford to be without, as it contains much new information of the greatest practical value. Medicines are here differently treated or considered under different heads from what they are in other works. But where there is such positiveness of contrast in the effects of the agents as will justify the inclusion of Digitalis under the head of Cardiac Stimulants, and Veratrum Viride under the head of Cardiac Sedatives, will puzzle the majority of physicians to determine. As a text book it is not equal to others which have long been before the profession, yet it is rich in original matter and in suggestions of thought. W. E. Q.

